# Notes on Nine Cases of Fracture at the Upper End of the Humerus

**Published:** 1907-07-13

**Authors:** Geo. S. Parkinson

**Affiliations:** Assistant House Surgeon, The Infirmary, Leicester


### RESIDENT MEDICAL OFFICERS' DEPARTMENT.

[Contributions to this Column are invited, and, if accepted, will be paid for.]

### NOTES ON NINE CASES OF FRACTURE AT THE UPPER END OF THE HUMERUS.

By GEO. S. PARKINSON, M.R.C.S., L.R.C.P., Assistant House Surgeon, The Infirmary, Leicester.

In the treatment of fractures complete immobilisation of the part for any length of time is now a thing of the past. Everyone is agreed that the best results are attained where early massage and movement have been adopted. This is more especially the case in fractures in the region of a joint, because, apart from the excessive formation of callus, fibrous adhesions may seriously impair function. In fractures involving the shoulder joint various forms of retentive apparatus have been used from time to time, the most common of which is, perhaps, the shoulder cap. This is the best apparatus when any is used, but it is not always necessary. With regard to other forms of splints, we are all aware that the prolonged use of splints may lead to a stiff joint, to say nothing of ischæmic contraction or pressure on nerves, leading to atrophy of muscles. With fractures of the upper end of the humerus, where the normal range of movement is great, any limitation is most detrimental.

The following notes have been taken on nine consecutive cases of fractured surgical neck of the humerus; in each a radiogram was taken to confirm the diagnosis. In no case was any splint or shoulder cap used, and in all the result was most favourable.

A list of the patients with their ages, occupations, and injuries is now appended.

| Sex | Age | Occupation | Injury |
|---|---|---|---|
| 1. Man ... | 39 | Mechanic ... | Fractured surgical neck |
| 2. Man ... | 32 | Mechanic ... | Fractured surgical neck |
| 3. Woman ... | 35 | House work | Impacted surgical neck with oblique fracture in upper ⅓. |
| 4. Boy ... | 11 | School ... | Separated epiphysis |
| 5. Woman ... | 65 | House work | Fractured surgical neck; had broken radius six years previously |
| 6. Man ... | 48 | Police Sergeant | Comminuted fracture of surgical neck |
| 7. Man ... | 54 | Traveller ... | Comminuted fracture of surgical neck |
| 8. Man ... | 39 | Shoe hand | Comminuted fracture of surgical neck |
| 9. Boy ... | 13 | — | Separated epiphysis |

### TREATMENT.

On the day of injury, lotio plumbi c̄ opio is applied to the injured parts, and a pad of wool is placed in the axilla, held in position by a three-inch bandage applied in the form of a spica; another piece of wool is placed in the opposite axilla to prevent chafing. The arm is then supported in a sling; this does not include the elbow, which is left free for purpose of extension. The arm is not bandaged to the side. On the following day lotio plumbi c̄ opio is again used, and a fresh pad placed in the axilla as before. On the third day very gentle massage is begun, the muscles round the injured joint being lightly rubbed while the arm remains in the sling. The arm is then taken out of the sling, carefully supported, and rotated through no more than a quarter of a circle; then the shoulder spica, pad, and sling are re-applied.

This treatment is continued for three weeks, the pad in the axilla being omitted at the end of a week. The patient comes up three times each week. On each occasion the normal movements of the shoulder joint are practised, the range of movement being increased at each visit. At least once in every week faradic current should be given, the motor points of the muscles being picked out with the electrode. At the end of three weeks the whole arm, including the elbow, is carried in a sling, and active movement permitted. Heavy massage is now of benefit, that is, kneading of the muscles combined with firm rubbing. At the same time, exercises should be ordered such as:—

1. Raising both arms from the sides, with elbow successively flexed and extended.
2. Bringing the arms straight out in front, palms of the hands together, and sweeping them back in a line with the shoulders against resistance.
3. Placing the hands on the head, behind the back, and on the opposite shoulder, with rotation of the humerus in the glenoid cavity.

Patients are given the B.P. lin. pot. iod. c̄. sapone to rub the shoulder with at home, and encouraged to use the injured arm as much as possible. This treatment lasts six or seven weeks.

Of the nine cases treated in the manner described the first four on the list could raise the arm nearly vertically above the head, all the other movements at the shoulder joint being equally free. The two mechanics returned to their work at the end of ten weeks, and were able to follow their trade. The boy aged 11 with the separated epiphysis made such a good recovery that, when moving both arms at the same time, several people were unable to state which had been the injured limb. The woman, though the head of the bone appeared to be fractured in two places, also got a very useful arm. In the early days of her treatment the deltoid showed some signs of atrophy, and the response to faradic current was sluggish, but under constant massage the muscle soon regained its tone. Of the next four, the woman of 65 years of age suffered from rheumatism, and severe movement was not practised; she, however, could raise the arm nearly to a right-angle with the side, was able to feed herself, and had some rotation. The other three cases were all very bad fractures, the radiogram showing marked comminution. In every case the hand could be placed on the head, behind the back, on the opposite shoulder, and a certain amount of rotation was present. The ninth case is a boy with a separated epiphysis; he is still under treatment, and progresses favourably.

These nine cases illustrate the fact that, to get union in fractures, complete immobilisation is unnecessary, and in many regions, as about the shoulder, inadvisable.

